# Negative Oxygen Ions Production by Superamphiphobic and Antibacterial TiO_2_/Cu_2_O Composite Film Anchored on Wooden Substrates

**DOI:** 10.1038/srep26055

**Published:** 2016-05-27

**Authors:** Likun Gao, Zhe Qiu, Wentao Gan, Xianxu Zhan, Jian Li, Tiangang Qiang

**Affiliations:** 1 Material Science and Engineering College, Northeast Forestry University, Harbin 150040, P.R. China; 2 Dehua TB New Decoration Material Co., Ltd, Huzhou, 313200, P.R. China

## Abstract

According to statistics, early in the 20th century, the proportion of positive and negative air ions on the earth is 1 : 1.2. However, after more than one century, the equilibrium state of the proportion had an obvious change, which the proportion of positive and negative air ions became 1.2 : 1, leading to a surrounding of positive air ions in human living environment. Therefore, it is urgent to adopt effective methods to improve the proportion of negative oxygen ions, which are known as “air vitamin”. In this study, negative oxygen ions production by the TiO_2_/Cu_2_O-treated wood under UV irradiation was first reported. Anatase TiO_2_ particles with Cu_2_O particles were doped on wooden substrates through a two-step method and further modification is employed to create remarkable superamphiphobic surface. The effect of Cu_2_O particles dopant on the negative oxygen ions production of the TiO_2_-treated wood was investigated. The results showed that the production of negative oxygen ions was drastically improved by doping with Cu_2_O particles under UV irradiation. The wood modified with TiO_2_/Cu_2_O composite film after hydrophobization is imparted with superamphiphobicity, antibacterial actions against Escherichia coli, and negative oxygen ions production under UV irradiation.

Nowadays, with the development of social and economic and the improvement of living standards, the human health awareness is also growing. Especially after the mechanism of the action of negative oxygen ions and its effects have been increasingly understood, the negative oxygen ions products will be developed as a kind of functional products^1^,^2^,^3^. As the oxygen molecule captured an electron, the oxygen molecule is defined as negative oxygen ion (O_2_^−^)^4^,^5^. The negative oxygen ions are also known as “air vitamin” due to its important meanings to human life activities. Meanwhile, the effects of negative oxygen ions on human health and ecological environment have been validated by domestic and foreign medical experts through clinical practices^6^,^7^. The researchers have found that negative oxygen ions could combine with bacteria, dust, smoke and some other positively charged particles in the atmosphere, and fall to the ground after gathering into balls, which would lead to the sterilization and the elimination of peculiar smell, that is, a purification of atmosphere. And the biological effects of superoxide (O_2_^−^) are considered in relation to the reactions leading to cell death and to the metabolism of certain endogenous compounds^8^. At the same time, O_2_^−^ production probably take place by surface ionization, which is identified as a viable ionization technique to have the potential to meet the requirements concerning ionization efficiency for the energy range of 10 eV to 1 keV within the limitations imposed by the resources (space, weight, powder, light, ect.) available on a proper surface^5^. Therefore, artificial method to achieve negative oxygen ions include UV irradiation, anion incentive, thermionic emission, corona discharge, charge separation, high-pressure water injection, tourmaline and other natural ores, and so on. In this paper, we adopt anion incentive method, which selected suitable photocatalyst irradiated by light to excite energy, and the excitation energy ionized the oxygen molecule to produce negative ions. At the same time, O_2_^−^ production probably takes place under the natural conditions as a result of gas ionization in the atmosphere.

So far, in most applications of photocatalyst, an n-type TiO_2_ semiconductor has been used^9^,^10^,^11^. Semiconductor materials are materials whose valence band and conduction band are separated by an energy gap or band gap. When the semiconductor material absorbs photons with energy equal or larger than its band gap, electrons in the valence band can be excited and migrate to the conduction band. For example, when TiO_2_ semiconductor materials with the forbidden bandgap of 3.2 eV are irradiated by light consisting of wavelengths shorter than 415 nm, the electron-hole pairs are produced after the migration of electrons. Then, the electron and hole react with water and oxygen, it would produce hydroxyl radicals (·OH) and superoxide (O_2_^−^). However, the recombination of the electron and the hole would happen, that is, the superoxide (O_2_^−^) is reduced. Therefore, in order to suppress the recombination of the electron and the hole and enhance the migration of charges, much attention has been focused on the modification of TiO_2_ by adding metal ions or oxides^12^,^13^,^14^. Among the metal ions and oxides, Cu_2_O is a nonstoichiometric p-type semiconductor with a direct forbidden bandgap of about 2.0 eV, whose component elements are inexpensive and abundantly available^15^,^16^. And doping Cu_2_O with TiO_2_ forming an n-p heterostructure is an effective way to enhance the photocatalytic activity of TiO_2_ photocatalyst.

As a natural material, wood is one of the most versatile and widely used structural materials for indoor and outdoor applications because of its many attractive properties, such as its low density, thermal expansion, renewability, and aesthetics^17^. Especially for indoor applications, such as floorboards and furniture, however, the process technology with the using of urea formaldehyde resin causes serious air pollution leading to human disease^18^. In addition, negative oxygen ions have a lot of advantages for environment including dust extraction, bacteriostasis and deodorization. Therefore, negative-ion wooden materials may have promising prospect in the future development of multifunctional materials.

Based on above considerations, in this paper, we imagine a TiO_2_/Cu_2_O composite coating on a wooden substrate, and the further modification with low surface energy of (heptadecafluoro-1,1,2,2-tetradecyl)trimethoxysilane is employed to create remarkable superamphiphobic surface. The wood decorated with TiO_2_/Cu_2_O composite film after hydrophobization might also be imparted with superamphiphobicity, antibacterial actions against Escherichia coli, and negative oxygen ions production under UV irradiation. Moreover, the mechanism of the negative oxygen ions production on TiO_2_/Cu_2_O-treated wood under UV irradiation was discussed in the paper.

## Results

**Figure 1:**
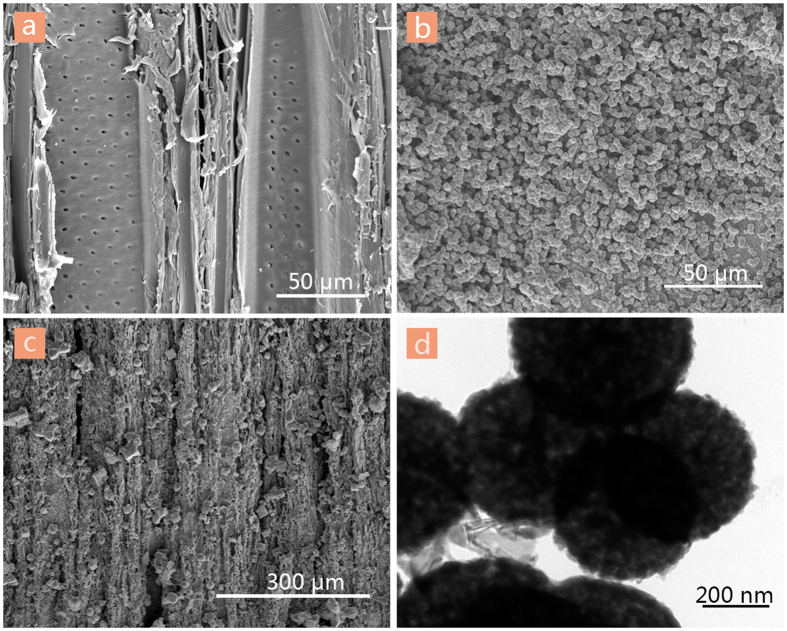
SEM images of the surfaces of (**a**) the original wood, (**b**) the TiO_2_-treated wood, and (**c**) the TiO_2_/Cu_2_O-treated wood. (**d**) TEM image of the TiO_2_/Cu_2_O-treated wood.

Field-emission scanning electron microscopy (FE-SEM) images in Fig. 1 show microstructural features of the procedure of the TiO_2_/Cu_2_O composite film deposited on the wood substrates. For the pristine wood (Fig. 1a), the surface is smooth and clean, but there are a few of pits, which indicates that the poplar wood is a type of heterogeneous and porous material. After low-temperature hydrothermal synthesis, the wood surface is covered by TiO_2_ microparticles with average diameter of 2.25 μm, as shown in Fig. 1b. Low magnification image in Fig. 1c shows that the TiO_2_/Cu_2_O composite layer is very dense, and the Cu_2_O particles spread all over the top surface of the TiO_2_-treated wood. The composite film replicates the grain structures with TiO_2_ particles and Cu_2_O particles. The transmission electron microscopy (TEM) image of the TiO_2_/Cu_2_O-treated wood in Fig. 1d shows that the film is formed by rough grains with average diameter of 0.60 μm. It is worth pointing out that the rough grains would significantly increase the water-repellent properties of the surfaces^19^.

**Figure 2:**
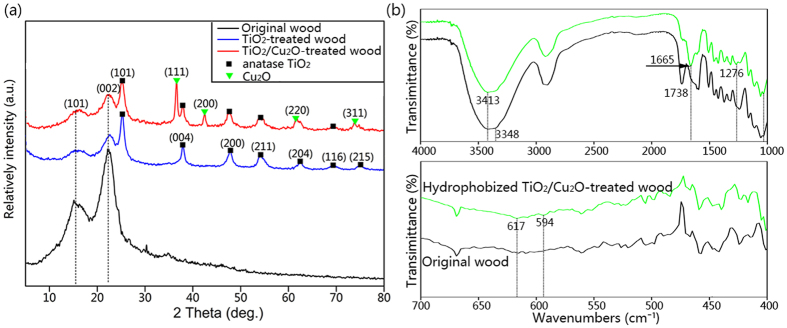
(**a**) XRD patterns of the original wood, the TiO_2_-treated wood, and the TiO_2_/Cu_2_O-treated wood. (**b**) FTIR spectra of the original wood and the hydrophobized TiO_2_/Cu_2_O-treated wood.

Figure 2a shows the XRD patterns of the original wood, the TiO_2_-treated wood and the TiO_2_/Cu_2_O-treated wood. For the original wood, there is no peak except the diffraction peaks at 14.8° and 22.5° belonging to the (101) and (002) crystal planes of cellulose in the wood, which could be observed in both the spectrum of the original wood and the treated wood samples^20^. It can be found that the diffraction peaks are well indexed to the standard diffraction pattern of anatase phase TiO_2_ (JCPDS file No. 21–1272) and the cuprite phase of Cu_2_O (JCPDS file No. 05–0667)^21^, indicating that the present synthesis strategy successfully achieves TiO_2_/Cu_2_O heterostructures with high crystallinity on wood substrate. After deposition with TiO_2_ particles, diffraction peaks at 25.2°, 38.0°, 47.8°, 54.2°, 62.5°, 68.8° and 74.9°, can be perfectly identified to (101), (004), (200), (211), (204), (116) and (215) crystal planes of anatase TiO_2_, respectively^22^. The red curve in Fig. 2a shows that all of the new diffraction peaks of the TiO_2_/Cu_2_O-treated wood center at 2*θ* = 36.4°, 42.3°, 61.6° and 73.8°, except the diffraction peaks of TiO_2_, are agree with (111), (200), (220) and (311) planes of pure cuprite Cu_2_O^17^.

Figure 2b shows FTIR spectra of the pristine wood and the hydrophobized TiO_2_/Cu_2_O-treated wood. The band at 3348 cm^−1^ corresponding to the stretching vibrations of OH groups in the wood (black curve) shifts to larger wavenumbers of 3413 cm^−1^ (green curve), and the intensity comparatively decreases, indicating that the hydrophilic groups (−OH) of the wood decreases in the hydrophobized TiO_2_/Cu_2_O-treated wood^23^. The band at 1738 cm^−1^ is attributed to the C=O stretching vibrations of carboxylic acid and acetyl group in hemicelluloses (black curve), while the band at 1665 cm^−1^ assigned to an conjugated carbonyl in lignin is observed (green curve). In Fig. 2b, the existence of C–F bonds in CF, CF_2_ or CF_3_ are located at 1276 cm^−1^ and 594 cm^−1 ^ 24, and the band at 617 cm^−1^ corresponds to stretching vibration of Cu(I)–O bond (optically active lattice vibration in the oxide)^25^,^26^. That is, the as-synthesized TiO_2_/Cu_2_O heterostructures on the wood surface contain Cu_2_O without CuO.

**Figure 3:**
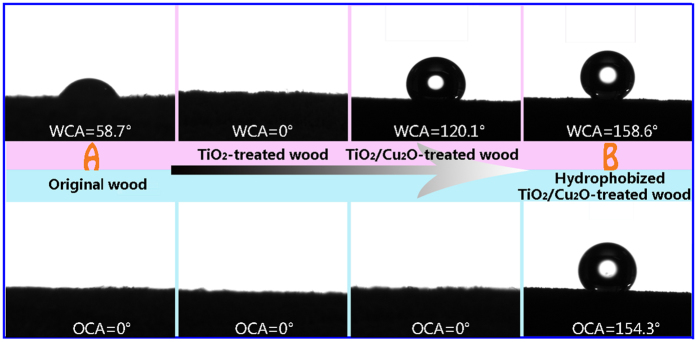
WCAs and OCAs of the original wood, the TiO_2_-treated wood, the TiO_2_/Cu_2_O-treated wood, and the hydrophobized TiO_2_/Cu_2_O-treated wood.

Figure 3 demonstrates the variation (from region A to region B) of the water contact angles and oil (hexadecane) contact angles of the original wood, the TiO_2_-treated wood, TiO_2_/Cu_2_O-treated wood, and the hydrophobized TiO_2_/Cu_2_O-treated wood. The original wood surface presents hydrophilicity with the WCA of 58.7° and superoleophilicity with the OCA of 0°. And the TiO_2_-treated wood possesses a superamphiphilic surface with both the WCA and the OCA of 0°. After coated with Cu_2_O particles, the wood surface converts into hydrophobic one with the WCA of 120.1°, while remains superoleophilic with the OCA of 0°. According to the literatures, higher treatment temperature levels had a likely round-shape of water droplets on the surface, however it is more flat shape on the original sample since the liquid rapidly absorbed and lead to small contact angle^27^,^28^,^29^. Surface of wood becomes smoother with increasing temperatures of heat treatment having better wettability expressed with larger contact angle value. Hemicellulose is greatly affected during the heat treatment and the organic acid is released from the degradation of the hemicellulose, which influences the cross-linking reduction in hydroxyl groups (OH−). Hydrophobicity of wood will also increase with decreasing of the (OH−) groups. Furthermore, it is known that the larger roughness and the lower surface energy of surfaces would lead to the hydrophobicity. After coating with Cu_2_O particles, the roughness of the surface became larger to produce a hydrophobic surface.

After further hydrophobization with FAS-17, the hydrophobicity of the wood surface is raised to superamphiphobicity, while the WCA and the OCA reach 158.6° and 154.3°, respectively. The results are in accordance with the FTIR results that the hydrophilic groups (–OH) of the wood decease in the hydrophobized TiO_2_/Cu_2_O-treated wood, that is, the liquid-repellent properties of wood surfaces are significantly increased.

**Figure 4:**
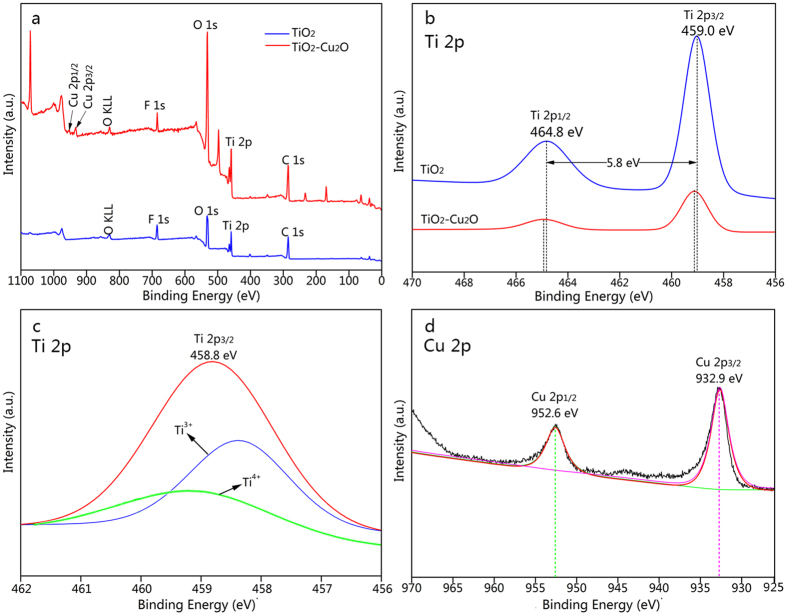
(**a**) Survey scan and (**b**) Ti 2p XPS spectra of the TiO_2_-treated wood and the TiO_2_/Cu_2_O-treated wood, (**c**) peaking-fitting results of Ti 2p XPS spectra of the TiO_2_/Cu_2_O-treated wood after UV light irradiation, (**d**) Cu 2p XPS spectra of the TiO_2_/Cu_2_O-treated wood.

Figure 4 ascertains more detailed information concerning the elemental and chemical state of the TiO_2_-treated wood and the TiO_2_/Cu_2_O-treated wood examined by using XPS. The fully scanned spectra (Fig. 4a) shows that the O, Ti and C elements exist on the surface of the pure TiO_2_-treated wood, while the Cu, O, Ti and C elements exist on the surface of the TiO_2_/Cu_2_O-treated wood. The C element can be ascribed to the wood substrate or the adventitious carbon–based contaminant.

From the peaking-fitting for the Ti 2p (Fig. 4b) in the TiO_2_-treated wood and the TiO_2_/Cu_2_O-treated wood, two peaks at binding energies of 459.0 eV and 464.8 eV corresponding to the Ti 2p_3/2_ and Ti 2p_1/2_ peaks can be observed. And the gap of 5.8 eV between the two peaks indicates the existence of the Ti^4+^ oxidation state^30^. However, the peak position for Ti 2p in the TiO_2_/Cu_2_O-treated wood shifts to a higher binding energy band than that in the pure TiO_2_-treated wood. This confirms a lower electron density of the Ti atoms after coated with Cu_2_O particles, and there is a strong interaction between Cu_2_O and TiO_2_ in the TiO_2_/Cu_2_O-treated wood. According to the standard binding energy of Ti 2p_3/2_, one located at 459.5 eV, usually attributed to a Ti^4+^ species, and the other located at 457.7 eV, assigned to a Ti^3+^ species. The binding energy of Ti 2p_3/2_ in the pure TiO_2_-treated wood and the TiO_2_/Cu_2_O-treated wood without UV light irradiation is 459.0 eV and 459.2 eV, respectively. The binding energy of Ti 2p_3/2_ shown in Fig. 4c is 458.8 eV, whose peak is much broader than that for the pure TiO_2_-treated wood and the TiO_2_/Cu_2_O-treated wood without UV light irradiation. The spectrum of Ti 2p_3/2_ of the TiO_2_/Cu_2_O-treated wood after UV light irradiation (Fig. 4c) is simulated with Gaussian simulation. The fitting peak at 458.2 eV is attributed to Ti^3+^, and that at 459.0 eV is attributed to Ti^4+^, respectively^31^. Thus, Ti^3+^ does exist in the TiO_2_/Cu_2_O-treated wood when it is irradiated under UV light. Certainly, the presence of Ti^3+^ oxide with narrow band gap and energy level located between the valence band and the conduction band of TiO_2_, may be advantageous to the higher photocatalytic activity of the TiO_2_/Cu_2_O heterostructures^32^.

As shown in Fig. 4d, it can be found that two main XPS peaks at 952.6 eV and 932.9 eV, which can be attributed to the double peaks for Cu 2p_3/2_ and Cu 2p_1/2_ of Cu_2_O, respectively, indicating that the oxidation state of Cu is +1^25^.

**Figure 5:**
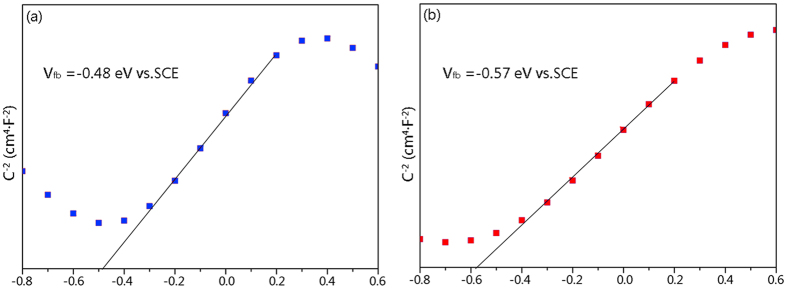
Mott-Schottky plots of (**a**) the pure TiO_2_-treated wood and (**b**) the TiO_2_/Cu_2_O-treated wood.

To investigate the band alignment and thus the charge transfer between TiO_2_ and Cu_2_O on the TiO_2_/Cu_2_O-treated wood, the Mott-Schottky (M-S) plots of the pure TiO_2_-treated wood and the TiO_2_/Cu_2_O-treated wood (Fig. 5) based on the following equation^33^:


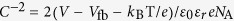


are used to determine their flat band potential *V*_fb_ and charge carrier density *N*_A_. Here, *C* is the space-charge capacitance of the semiconductor; *ε*_0_ is the permittivity in vacuum (*ε*_0_ = 8.85× 10^−14^ F/cm); *ε*_r_ is the dielectric constant; *V* is the applied potential; T is the temperature and *k*_B_ is Boltzmann constant (*k*_B_ = 1.38 × 10^−23^ J/K). From the linear fit of *C*^−2^ versus *V*, *V*_fb_ for the pure TiO_2_-treated wood and the TiO_2_/Cu_2_O-treated wood is obtained to be −0.48 eV and −0.57 eV νs. SCE, respectively. Therefore, it can be observed a negative shift of the flat band potential in Fig. 5b that indicates the sample after coated with Cu_2_O particles needs to cross smaller potential barrier and is beneficial to the production of photogenerated electrons and holes.

**Figure 6:**
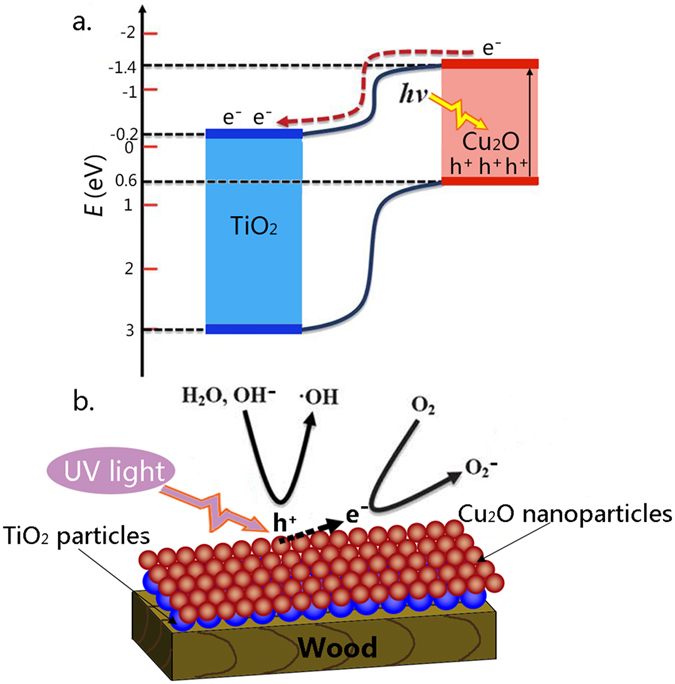
(**a**) Illustration of the photocatalysis mechanism at the TiO_2_/Cu_2_O heterostructures, and (**b**) possible scheme for negative oxygen ions production on the TiO_2_/Cu_2_O-treated wood under UV irradiation.

Considering the respective band gaps of 3.2 eV and 2.0 eV for TiO_2_ and Cu_2_O, the band alignment diagram of the TiO_2_/Cu_2_O heterostructures is shown in Fig. 6a. When TiO_2_ and Cu_2_O came into contact, an n-p junction formed at their interface. It is well known that the conduction band of TiO_2_ is about −0.2 eV, and the potential of Cu_2_O conduction band is −1.4 eV. The photogenerated electrons from the conduction band of Cu_2_O were captured by Ti^4+^ ions in TiO_2_, and Ti^4+^ ions were further reduced to Ti^3+^ ions. The Ti^3+^ ions have a long lifetime and bear the photogenerated electrons as a form of energy^34^. The electron-transfer process is shown in Eqs (1) and (2):


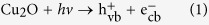



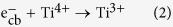


While on the TiO_2_/Cu_2_O heterostructures, the ·OH and H^+^ came from the indirect oxidization of the adsorbed water or hydroxyl groups at the positive holes^35^. Meanwhile, the negative oxygen ions (O_2_^−^) are produced through the reaction between the negative electron and the oxygen molecules. Moreover, the energy stored in the Ti^3+^, may promote a trapping of electrons leading to produce negative oxygen ions under the natural conditions^34^. Under UV irradiation, the negative oxygen ions production process (Fig. 6b) is shown in Eqs (3, 4, 5):


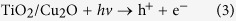



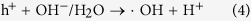



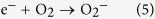


**Figure 7:**
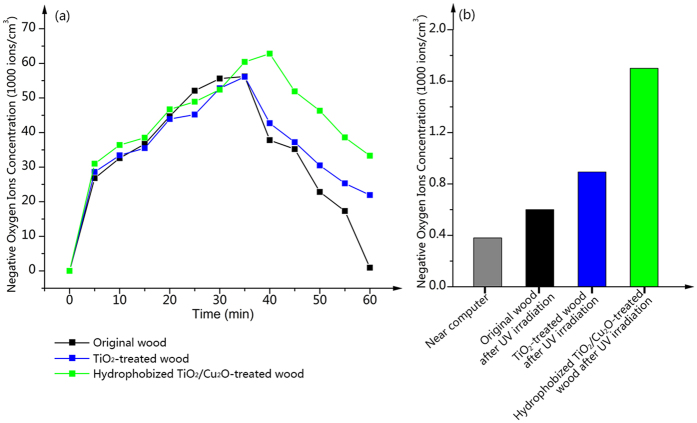
(**a**) Negative oxygen ions concentrations in the obturator when UV irradiated the samples surfaces. (**b**) Negative oxygen ions concentration in the environment near computer and negative oxygen ions concentrations in the obturator after UV irradiated the samples surfaces for 60 minutes.

In order to investigate the negative oxygen ions production in the obturator when UV irradiated the samples surfaces, the negative oxygen ions concentrations in the obturator for every 5 minutes are depicted in Fig. 7a. Firstly, the initial negative oxygen ions concentrations in the obturator for the three samples are 0 ions/cm^3^. It is obvious that, for both the TiO_2_-treated wood and the hydrophobized TiO_2_/Cu_2_O-treated wood, the negative oxygen ions concentrations are dramatically increased, after 35 or 40 minutes irradiation, the concentrations is decreased but still superior to that of the original wood. The blame for falling lays on that the negative oxygen ions will not increase indefinitely, and the negative oxygen ions concentration in the nature locates between 700 and 4000 ions/cm^3^, which may ascribed to the neutralization of the positive and negative ions, and the inhibition effect of the negative ions in the atmosphere to maintain a level when the negative ions concentration saturates to a certain extent.

Figure 7b presents the negative oxygen ions concentration in the environment near computer and the negative oxygen ions concentrations in the obturator after UV irradiated the samples surfaces for 60 minutes. The negative oxygen ions concentration in the environment near computer is achieved by calculating the average of the total amount concentration of every 5 minutes in 60 minutes. As for the environment near computer, the negative oxygen ions concentration in the atmosphere is 380 ions/cm^3^, which may be the reason that causes physiological barrier and severe illness. And for the original wood and the TiO_2_-treated wood after irradiation, the negative oxygen ions concentrations in the obturator are 600 and 900 ions/cm^3^, respectively. Whereas for the hydrophobized TiO_2_/Cu_2_O-treated wood, it exhibits a much higher concentrations of 1700 ions/cm^3^, indicating the photocatalytic activity and negative oxygen ions production efficiency of the hydrophobized TiO_2_/Cu_2_O-treated wood is significantly improved due to the loading of the Cu_2_O particles onto the surface of the TiO_2_-treated wood. According to the regulation of World Health Organization, when the concentration of negative oxygen ions in the air is more than 1000 ∼ 1500 ions/cm^3^, the air is regarded as the fresh air^36^. Thus, the air including negative oxygen ions produced by the hydrophobized TiO_2_/Cu_2_O-treated wood under UV irradiation is up to the standard of the fresh air.

**Figure 8:**
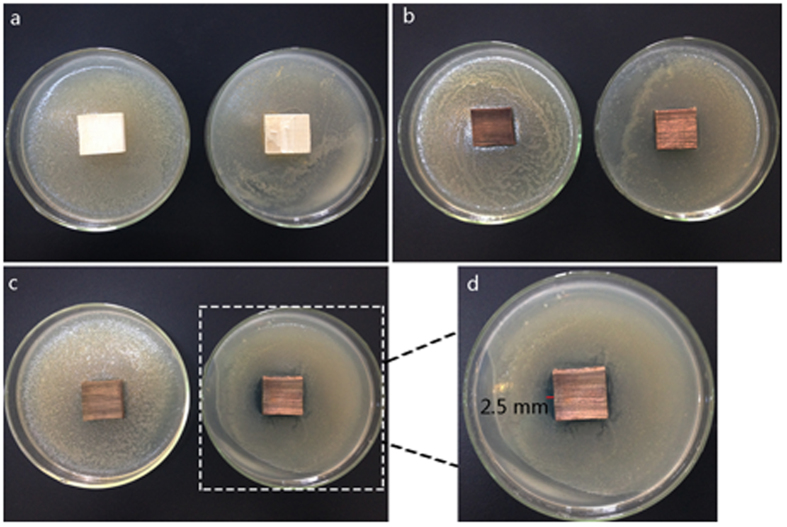
Antibacterial activity of (**a**) the TiO_2_-treated wood, (**b**) the Cu_2_O-treated wood and (**c**) the hydrophobized TiO_2_/Cu_2_O-treated wood in Staphylococcus aureus and Escherichia coli, respectively, and (**d**) the magnified picture of the hydrophobized Ag/TiO_2_–coated wood in Escherichia coli.

Antibacterial activities of wood samples are determined in terms of inhibition zone formed on agar medium. Figure 8a,b present that the TiO_2_-treated wood and the Cu_2_O-treated wood, which are used as control groups, do not show any antibacterial activity for both Escherichia coli and Staphylococcus aureus. In Fig. 8c, it could be seen that for Escherichia coli, the hydrophobized TiO_2_/Cu_2_O-treated wood placed on the bacteria−inoculated surfaces kill all the bacteria under and around them, while it has no antibacterial activity for Staphylococcus aureus. It can be observed that the distinct zones of inhibition (clear areas with no bacterial growth) around the wood samples for Escherichia coli (Fig. 8d). For Escherichia coli, the width of the inhibition zone around the hydrophobized TiO_2_/Cu_2_O-treated wood is approximately 2.5 mm. The observed zone of inhibition is a result of the performance of the negative oxygen ions production of the hydrophobized TiO_2_/Cu_2_O film anchored on the wood surface into the surrounding aqueous medium, which further proves that the hydrophobized TiO_2_/Cu_2_O-treated wood could produce negative oxygen ions not only under UV light but also under the natural conditions. In other words, the presences of the inhibition zone clearly indicate that the mechanism of the biocidal action of the wood is owing to the effect of negative oxygen ions.

## Discussion

The enhanced photocatalytic properties and excellent multifunctional performances of the hydrophobized TiO_2_/Cu_2_O-treated wood were probably attributed to the following four points: (a) The anatase TiO_2_ particles settled onto the wood surfaces and formed a dense film, which offered large n-p heterojunction interface area when in combination with Cu_2_O film. The treatment at high temperature and the rough TiO_2_/Cu_2_O grains would significantly increase the water-repellent properties of the surfaces; (b) After modification with FAS-17, the amphiphilic wood surface transformed into superamphiphobic one with the WCA of 158.6° and OCA of 154.3°; (c) The step-wise energy band structure facilitated the photogenerated electrons from the conduction band of Cu_2_O were captured by Ti^4+^ ions in TiO_2_ while promoted Ti^4+^ ions further to reduce to Ti^3+^ ions. The presence of Ti^3+^ ions led to a higher photocatalytic activity and even extended the photo-response from UV to the visible light. Moreover, the energy stored in the Ti^3+^, may promote a trapping of electrons leading to produce negative oxygen ions under the natural conditions.; (d) The introduction of Cu_2_O significantly improved the photocatalysis efficiency of the TiO_2_-treated wood, so that the air including negative oxygen ions produced by the hydrophobized TiO_2_/Cu_2_O-treated wood after UV irradiation is up to the standard of the fresh air; (e) Compared with the pure TiO_2_-treated wood and the pure Cu_2_O-treated wood, the hydrophobized TiO_2_/Cu_2_O-treated wood possesses antibacterial activity for Escherichia coli, which may be due to the biocidal action of the negative oxygen ions.

In brief, the TiO_2_/Cu_2_O n-p heterostructure was successfully fabricated anchored on the wood surface, and after further modification with FAS-17 the wood surface transformed into superamphiphobic. The results demonstrated the doped Cu_2_O particles resulted in a higher photocatalytic activity, which the final produced negative oxygen ions after UV irradiation was up to the standard of the fresh air. Moreover, the presence of Ti^3+^ ions trapped the electrons leading to produce negative oxygen ions under the natural conditions, so that the hydrophobized TiO_2_/Cu_2_O-treated wood showed a good antibacterial activity for Escherichia coli. Moreover, the excellent superamphiphobicity, antibacterial activity and ability of negative oxygen ions production suggested that such heterostructured film anchored on wood surface is a potential candidate for advanced multifunctional materials, maybe for the extended field of medical or environment purification.

## Methods

### Materials

All chemicals supplied by Shanghai Boyle Chemical Company, Limited were of analytical reagent-grade quality and used without further purification. Deionized water was used throughout the study. Wood blocks of 20 mm (R) × 20 mm (T) × 30 mm (L) were obtained from the sapwood sections of poplar wood (*Populus ussuriensis* Kom), which is one of the most common tree species in the northeast of China. The wood specimens were oven-dried (24 h, 103 ± 2 °C) to constant weight after ultrasonically rinsing in deionized water for 30 min, and oven-dried weight were determined.

### Preparation of anatase TiO_2_ film on the wood surface

Ammonium fluorotitanate (0.4 M) and boric acid (1.2 M) were dissolved in distilled water at room temperature under vigorous magnetic stirring. Then, a solution of 0.3 M hydrochloric acid was added until the pH reached approximately 3. Then, 75 mL of the adjusted solution was transferred into a 100 mL Teflon container. Wood specimens were subsequently placed into the above reaction solution, separately. The autoclave was sealed and maintained at 90 °C for 5 h, then allowed to naturally cool to room temperature. Finally, the prepared samples were removed from the solution, ultrasonically rinsed with deionized water for 3 times, and dried at 45 °C for more than 24 h in vacuum. Thus, the TiO_2_-treated wood was obtained.

### Coating the TiO_2_-treated wood surface with Cu_2_O particles

Cu(CH_3_COO)_2_·H_2_O (0.1 M) was dissolved in deionized water, then D-glucose (0.1 M) and PVP (K-30, 0.3 g) were added under continued stirring. After 1 h, the above solution was transferred into a 50 mL Teflon-lined autoclave, and the TiO_2_-treated wood was immersed in the solution. The autoclave was kept in a vacuum oven at 180 °C for 2 h. After cooling to room temperature in air, the wood loaded with Cu_2_O was removed, washed several times with deionized water and ethanol, and dried at 45 °C for more than 24 h in vacuum. Thus, the TiO_2_/Cu_2_O-treated wood was obtained.

### Hydrophobization of wood surfaces with FAS-17

A methyl alcohol solution of (heptadecafluoro-1,1,2,2-tetradecyl)trimethoxysilane CF_3_(CF_2_)_7_CH_2_CH_2_Si(OCH_3_)_3_, hereafter denoted as FAS-17) was hydrolyzed by the addition of a 3 fold-molar excess of water at room temperature. Then, 75 mL of the adjusted solution was transferred into a 100 mL Teflon container. The TiO_2_/Cu_2_O-treated wood samples were subsequently placed into the above reaction solution. The autoclave was sealed and maintained at 75 °C for 5 h, then allowed to naturally cool to room temperature. Subsequently, the samples were washed with ethyl alcohol to remove any residual chemicals and allowed to dry in air at room temperature, and they were then dried at 45 °C for more than 24 h in vacuum. Thus, a superamphiphobic wood surface was obtained.

### Characterization

The morphology of the wood surfaces was observed through field emission scanning electron microscopy (SEM, Quanta 200, FEI, Holland) operating at 12.5 kV. The transmission electron microscopy (TEM) experiment was performed on a Tecnai G20 electron microscope (FEI, USA) with an acceleration voltage of 200 kV. Carbon-coated copper grids were used as the sample holders. X-ray diffraction (XRD, Bruker D8 Advance, Germany) was employed to analyze the crystal structures of all samples applying graphite monochromatic with Cu Kα radiation (λ = 1.5418 Å) in the 2θ range from 5° to 80°and a position-sensitive detector using a step size of 0.02° and a scan rate of 4° min^−1^. FTIR spectra were obtained on KBr tablets and recorded using a Magna-IR 560 spectrometer (Nicolet) with a resolution of 4 cm^−1^ by scanning the region between 4000 and 400 cm^−1^. Water contact angles (WCAs) and oil (hexadecane) contact angles (hereafter defined as OCAs) were measured on an OCA40 contact angle system (Dataphysics, Germany) at room temperature. In each measurement, a 5 μL droplet of deionized water was injected onto the surfaces of the wood samples and the contact angles were measured at five different points of each sample. The final values of the contact angles were obtained as an average of five measurements. Further evidence for the composition of the product was inferred from the X-ray photoelectron spectroscopy (XPS, Thermo ESCALAB 250XI, USA), using an ESCALab MKII X-ray photoelectron spectrometer with Mg-Kα X-rays as the excitation source. The flat band potentials of the samples were evaluated with a standard electrochemical workstation (CHI660C) in the three-electrode configuration. The counter electrode was a Pt wire and the reference electrode was saturated calomel electrode (SCE). 1.0 M Na_2_SO_4_ solution buffered at pH 4.9 was used as the electrolyte. In the electrochemical impedance spectroscopy (EIS) measurement, the fixed frequency of the M-S plots was 10^3^ Hz.

### Antibacterial tests

Antimicrobial tests were conducted by the bacterial inhibition ring method (agar plate diffusion test/CEN/TC 248 WG 13) and the reduction of bacterial growth test (EN ISO 20743:2007 Transfer Method). The antibacterial activity of the hydrophobized TiO_2_/Cu_2_O-treated wood was evaluated against Escherichia coli (ATCC 25923) and Staphylococcus aureus (ATCC 25922). The evaluation of the test was as the followings. A mixture of nutrient broth and nutrient agar in 1 L distilled water at pH 7.2 as well as the empty Petri plates were autoclaved. The agar medium was then cast into the Petri plates and cooled in laminar airflow. Approximately 10^5^ colony-forming units of each bacterium were inoculated on plates, and then each wood samples was planted onto the agar plates. All the plates were incubated at 37 °C for 24 h, following which the zone of inhibition was measured.

### Negative oxygen ions production tests under UV irradiation

The experiments were performed in an obturator with the size of 500 mm (L) × 300 mm (W) × 300 mm (H). A UV light with a wavelength of 365 nm and light intensity of approximately 1.5 mW/cm^2^ installed in the open central region was used. Negative oxygen ions concentration (D_a_) in the obturator was determined by the AIC1000 air anion counter with the resolution ratio of 10 ions/cm^3^ at 5 min intervals. Initially, the air anion counter must be re-zeroed and keep the numerical value constant for 5 seconds before each test. Then, the UV light was turned on to irradiate the sample. Each set of negative oxygen ions production under UV irradiation lasted for 60 min.

## Additional Information

**How to cite this article**: Gao, L. *et al.* Negative Oxygen Ions Production by Superamphiphobic and Antibacterial TiO_2_/Cu_2_O Composite Film Anchored on Wooden Substrates. *Sci. Rep.* **6**, 26055; doi: 10.1038/srep26055 (2016).
